# Myonuclear content and domain size in small versus larger muscle fibres in response to 12 weeks of resistance exercise training in older adults

**DOI:** 10.1111/apha.13599

**Published:** 2020-12-20

**Authors:** Tim Snijders, Andy M. Holwerda, Luc J. C. van Loon, Lex B. Verdijk

**Affiliations:** ^1^ Human Biology School of Nutrition and Translational Research in Metabolism (NUTRIM) Maastricht University Maastricht The Netherlands

**Keywords:** growth, human, hypertrophy, muscle, myonuclear domain size

## Abstract

**Aim:**

To assess the relation between muscle fibre hypertrophy and myonuclear accretion in relatively small and large muscle fibre size clusters following prolonged resistance exercise training in older adults.

**Methods:**

Muscle biopsies were collected before and after 12 weeks of resistance exercise training in 40 healthy, older men (70 ± 3 years). All muscle fibres were ordered by size and categorized in four muscle fibre size clusters: ‘Small’: 2000‐3999 µm^2^, ‘Moderate’: 4000‐5999 µm^2^, ‘Large’: 6000‐7999 µm^2^ and ‘Largest’: 8000‐9999 µm^2^. Changes in muscle fibre size cluster distribution were related to changes in muscle fibre size, myonuclear content and myonuclear domain size.

**Results:**

With training, the percentage of muscle fibres decreased in the Small (from 23 ± 12 to 17 ± 14%, *P* < .01) and increased in the Largest (from 11 ± 8 to 15 ± 10%, *P* < .01) muscle fibre size clusters. The decline in the percentage of Small muscle fibres was accompanied by an increase in overall myonuclear domain size (*r* = −.466, *P* = .002) and myonuclear content (*r* = −.390, *P* = .013). In contrast, the increase in the percentage of the Largest muscle fibres was accompanied by an overall increase in myonuclear content (*r* = .616, *P* < .001), but not in domain size.

**Conclusion:**

Prolonged resistance‐type exercise training induces a decline in the percentage of small as well as an increase in the percentage of the largest muscle fibres in older adults. Whereas the change in the percentage of small fibres is best predicted by an increase in overall myonuclear domain size, the change in the percentage of the largest fibres is associated with an overall increase in myonuclear content.

## 1 INTRODUCTION

Skeletal muscle fibres are large multinucleated cells, with every muscle fibre containing hundreds to thousands of nuclei.^1^ Myonuclei are not randomly scattered across the muscle fibre, but specifically positioned to minimize transport distances. Ever since the 19th century, it has been hypothesized that every nucleus holds jurisdiction over a certain volume of the muscle fibre cytoplasm, initially referred to as the ‘karyoplasmatic’ ratio.^2^ More recently, the division of a muscle fibre into evenly distributed domains has been referred to as the ‘DNA unit’ or ‘myonuclear domain’.^3^, ^4^ Within the concept of the ‘myonuclear domain theory’, each myonucleus in a fibre is suggested to control the mRNA transcription of a certain surrounding volume of cytoplasm called the myonuclear domain.^4^, ^5^ The theory postulates that a linear relationship exists between total myonuclear number and muscle fibre size and/or volume.^6^ Furthermore, the myonuclear domain is kept (relatively) constant by adding additional nuclei (supplied by muscle satellite cells) during muscle fibre hypertrophy and nuclear loss (by apoptosis) during muscle fibre atrophy.^6^ However, whether myonuclei are truly lost during muscle fibre atrophy remains a highly debated topic.^7^, ^8^, ^9^, ^10^, ^11^ Originally, it was suggested that muscle fibre hypertrophy must exceed a certain threshold beyond which additional myonuclei are required,^12^, ^13^ a concept referred to as the ‘myonuclear domain ceiling’. In accordance, muscle fibres with a relatively low myonuclear domain size (ie large number of myonuclei relative to muscle fibre size) would theoretically have a greater growth potential when compared with muscle fibres with a large myonuclear domain size. Although this hypothesis has been confirmed within some animal models and has been framed as part of the ‘muscle memory’ paradigm,^14^, ^15^ such an association has never been observed in human skeletal muscle tissue.

To date, most studies that investigate the muscle fibre growth response in light of the myonuclear domain theory, typically report mean changes in the number of myonuclei, domain size and fibre cross‐sectional area (CSA) of the muscle fibres identified in muscle biopsy samples taken before and after prolonged exercise training.^8^, ^23^ However, muscle fibre sizes can be quite heterogeneously divided within a muscle biopsy sample, particularly in older adults who suffer from the development of age‐related sarcopenia.^24^ Brack et al^25^ were the first to introduce muscle fibre size‐dependent cluster analysis of myonuclear content and domain size and reported an age‐related reduction in the number of myonuclei in mice that mainly occurred in larger muscle fibres. Similar results were later reported by Karlsen et al^26^ showing that the myonuclear domain size is disproportionally small in smaller when compared with larger muscle fibres in human muscle biopsy samples. According to the myonuclear domain ceiling theory, this would indicate that small muscle fibres, with a disproportionally smaller myonuclear domain size, would have a greater capacity for muscle fibre hypertrophy when compared with larger muscle fibres. This may be of particular relevance in older adults who, as a result of age‐related muscle fibre atrophy, exhibit a relatively large number of small muscle fibres with disproportional small myonuclear domain sizes. However, in older adults the relative small myonuclear domain size in the smallest fibres may also reflect an age‐related reduction in myonuclear efficiency, which could potentially also explain the atrophy of these particular muscle fibres. Investigating the changes in myonuclear domain size within different muscle fibre size clusters could, therefore, provide more insight in the regulation of myonuclear content and the proposed necessity of myonuclear accretion during muscle fibre hypertrophy following prolonged exercise training in older adults. Karlsen et al^27^ were the first who used this approach to evaluate the muscle fibre hypertrophy and myonuclear accretion response in muscle biopsy samples taken from very old adults (83‐94 years) before and after 12 weeks of resistance exercise training. However, the lack of muscle fibre hypertrophy observed during the exercise training programme limited the authors’ ability to draw firm conclusions. In the present study, we re‐analysed muscle biopsy samples from a previously published study performed in our laboratory,^28^ in which significant muscle fibre hypertrophy was shown in 40 healthy, older men following 12 weeks of resistance exercise training. We used a muscle fibre size cluster approach to test the hypothesis that the overall muscle fibre hypertrophy response observed following 12 weeks of resistance exercise training was mainly associated with a decline in the proportion of small muscle fibres. Furthermore, we hypothesized that the exercise training‐induced decline in the proportion of small muscle fibres was mainly associated with an increase in myonuclear domain size and not myonuclear content.

## 2 RESULTS

### 2.1 Muscle strength and body composition

In the original study,^28^ subjects were randomly provided either a protein or placebo supplement throughout the 12‐week exercise training programme. As no difference was observed in the change in muscle strength and body composition over time between the placebo and protein‐supplemented group, all subsequent analyses were performed with all subjects in one group. In response to 12 weeks of resistance exercise training, a significant increase was observed in 1RM leg muscle strength assessed on both leg press (from 86 ± 14 to 103 ± 16 kg, *P* < .01) and leg extension (from 159 ± 24 to 184 ± 30 kg, *P* < .01). Whole body as well as leg lean tissue mass increased significantly over time (both *P* < .01). Furthermore, Quadriceps muscle CSA increased (7 ± 5%) significantly in response to the resistance exercise training programme (*P* < .01, Table 1).

**TABLE 1:** Lean mass, *M Quadriceps* cross‐sectional area (CSA) and muscle fibre characteristics before (pre) and after (post) 12 wk of resistance exercise training in healthy, older men

	Pre	Post
Whole body lean mass (kg)	59.7 ± 5.4	61.0 ± 5.9*
Leg lean mass (kg)	13.2 ± 1.4	13.6 ± 1.4*
*M Quadriceps* CSA (mm^2^)	6796 ± 700	7268 ± 779*
Muscle fibre CSA (µm^2^)	5746 ± 1419	6397 ± 1527*
Myonuclear content (per fibre)	3.38 ± 0.65	3.65 ± 0.69*
Myonuclear domain size (µm^2^)	1707 ± 326	1769 ± 355
Satellite cell content (per fibre)	0.064 ± 0.031	0.069 ± 0.029

Note

Data represent mean ± SD.

Abbreviation: CSA, cross‐sectional area.

* Significantly different compared with Pre, *P* < .05.

### 2.2 Average muscle fibre characteristics

No difference was observed in the change in muscle fibre size, satellite cell/myonuclear content or myonuclear domain size between the placebo or protein‐supplemented group (see Table S1). Hence, subsequent analyses were performed with all subjects in one group. Significant increases in muscle fibre CSA (16 ± 33%, *P* < .01) and myonuclear content (10 ± 22%, *P* < .01) were observed in response to 12 weeks of resistance exercise training (Table 1). In contrast, no significant changes over time were observed for both myonuclear domain size and satellite cell content (Table 1). Pearson correlation analyses showed a significant correlation between baseline muscle fibre CSA and myonuclear content (*r* = .748, *P* < .001, Figure S1A), myonuclear domain size (*r* = .559, *P* < .001, Figure S1B) and satellite cell content (*r* = .388, *P* < .001, Figure S1C). A significant correlation was observed between delta muscle fibre CSA and delta myonuclear content (*r* = .612, *P* < .001, Figure S1D). Finally, a significant correlation was observed between delta myonuclear content and delta satellite cell content (*r* = .320, *P* = .044).

### 2.3 Muscle fibres size clusters

**FIGURE 1:**
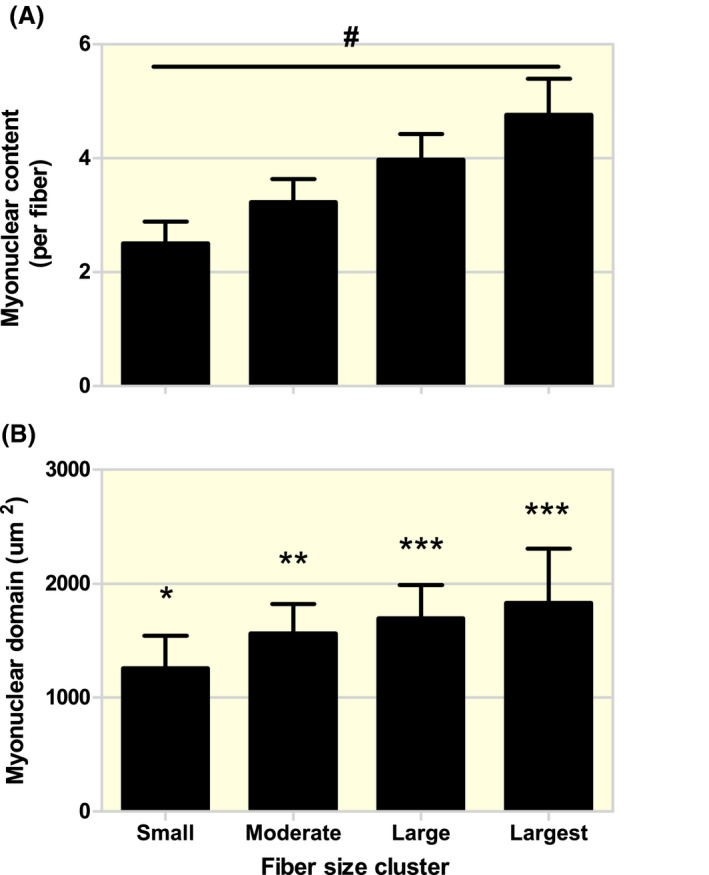
Baseline myonuclear content (A) and domain size (B) within the different muscle fibre size clusters. Small: cluster with fibres between 2000 and 3999 µm^2^, Moderate: cluster with fibres between 4000 and 5999 µm^2^, Large: cluster with fibres between 6000 and 7999 µm^2^, Largest: cluster with fibres between 8000 and 9999 µm^2^. Data are expressed as Mean ± SD. ^#^All groups are significantly different from each other, *P* < .05. *Significantly different compared with Moderate, Large and Largest, *P* < .05. **Significantly different compared with Small, Large and Largest, *P* < .05. ***Significantly different compared with Small and Moderate, *P* < .05

**FIGURE 2:**
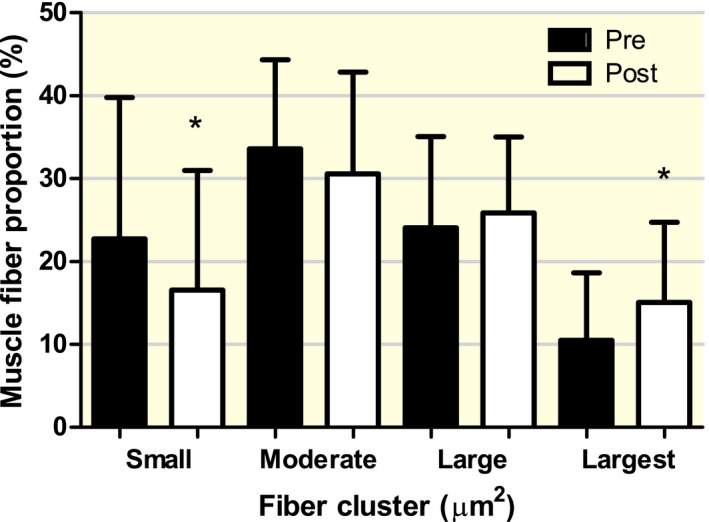
Proportion of muscle fibres located within the “Small” (2000 ‐ 3999 µm^2^), “Moderate” (4000 ‐ 5999 µm^2^), “Large” (6000 ‐ 7999 µm^2^) and “Largest” (8000 ‐ 9999 µm^2^) muscle fibre size clusters before (pre) and after (post) 12 wk of resistance exercise training in healthy, older men. Data are expressed as Mean ± SD. *Significantly different compared with Pre, *P* < .01

At baseline, myonuclear content was significantly different between all the different muscle fibre size clusters (Figure 1A). In addition, baseline myonuclear domain size was significantly different between all different muscle fibre size clusters, with the exception of the Large versus the Largest cluster (Figure 1B). For muscle fibre size distribution, a significant *fibre size cluster* × *time* interaction was observed (*P* < .01). Subsequent analysis showed a significant change in the percentage of muscle fibres in the Small (from 23 ± 12 to 17 ± 14%, *P* < .01) and Largest (from 11 ± 8 to 15 ± 10%, *P* < .01) muscle fibre size clusters over time, with no changes in the Moderate and Large clusters (Figure 2).

A significant negative correlation was observed between baseline percentage of muscle fibres located in the Small fibre size cluster and baseline muscle Quadriceps CSA (*r* = −.396) and 1RM leg press (*r* = −.457, both *P* < .05; Figure S2A‐C). Furthermore, a significant positive correlation was found for baseline percentage of muscle fibres located in the Largest fibre size cluster and baseline muscle Quadriceps CSA (*r* = .327) and 1RM leg press (*r* = .511, both *P* < .05; Figure S2B‐D).

**FIGURE 3:**
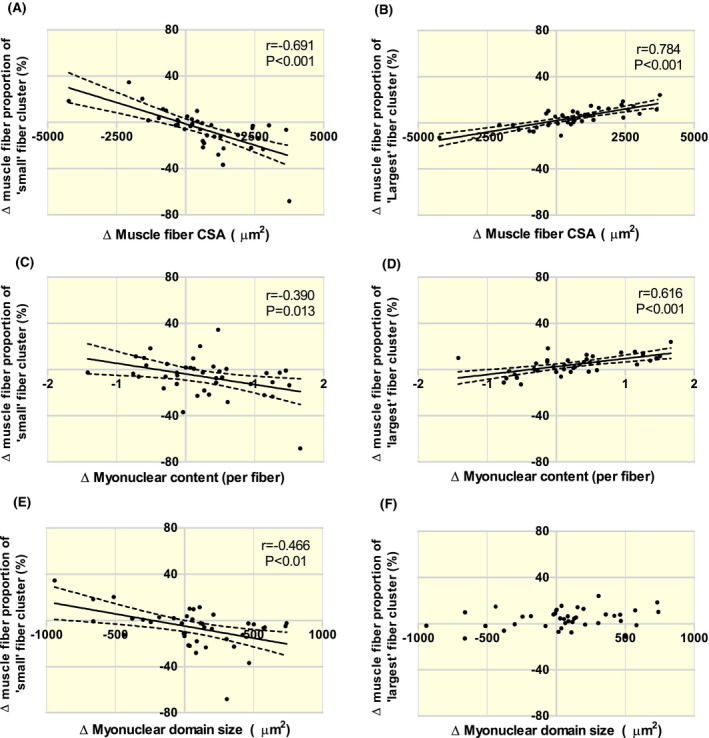
Scatter plots of the correlation analyses (Pearson's *r*, n = 40) between the changes in muscle fibre cross‐sectional area (CSA) and changes in the percentage of muscle fibres within the Small” (2000‐3999 µm^2^) (A, C and E) and “Largest” (8000‐9999 µm^2^) (B, D and E) muscle fibre size clusters in response to 12 wk of resistance exercise training. Solid line: regression line. Dotted line: 95% confidence interval

Next, we analyzed the correlation coefficients between the overall changes in muscle fibre size, myonuclear content, myonuclear domain size and change in muscle fibre percentage in the ‘Small’, ‘Moderate’, ‘Large’ and ‘Largest’ muscle fibre size clusters, respectively, in response to the 12 weeks of resistance exercise training. First, we observed that a larger decline in the percentage of muscle fibres located in the Small muscle fibre size cluster was associated with a larger overall increase in muscle fibre CSA following the 12 weeks of resistance exercise training (Figure 3A; *r* = −.691, *P* < .001). No significant and a relatively weak, but significant (*r* = .344, *P* = .03) correlation was observed between the change in the percentage of muscle fibres located within the Moderate and Large muscle fibre size clusters, respectively, and the overall increase in muscle fibre CSA. In contrast, we observed that a larger increase in the percentage of muscle fibres located within the Largest muscle fibre size cluster was associated with a larger overall increase in muscle fibre CSA following exercise training (Figure 3B; *r* = .784, *P* < .001)).

A significant (moderate) correlation was observed, showing that an increase in myonuclear content over time was associated with a decline in muscle fibre percentage located within the Small and Moderate muscle fibre size clusters (*r* = −.390 Figure 3C and *r* = −.358, both *P* < .025, respectively). Whereas no correlation was observed for the Large muscle fibre size cluster, a strong correlation was observed for the Largest cluster, showing that an increase in myonuclear content was associated with an increase in muscle fibre percentage located within the Largest muscle fibre size cluster (*r* = .616, *P* < .001, Figure 2D). When considering the change in myonuclear domain size over time, a significant (moderate) correlation was observed with the Small muscle fibre size cluster, indicating that an overall increase in myonuclear domain size was associated with a decline in muscle fibre percentage located within the Small cluster (*r* = −.466, *P* < .01, Figure 3E). No correlations were observed between the changes in myonuclear domain size over time and the changes in muscle fibre proportion located within the Moderate, Large or Largest (Figure 3F) muscle fibre size clusters. In addition, changes in the satellite cell content in response to exercise were not associated with changes in the percentage of fibres in any muscle fibre size cluster.

### 2.4 Linear regression modelling

Backward stepwise linear regression analysis was performed to assess whether changes in the percentage of muscle fibres per fibre size cluster (ie Small, Moderate, Large, Largest) were predictive of overall changes in muscle fibre CSA, myonuclear content and domain size following the 12‐week intervention period (See also Tables S3, S4 and S5). A decline in the percentage of muscle fibres in the Small fibre size cluster (*B* = −3133, standardized *B* = −0.335, *P* < .05) and an increase in the percentage of muscle fibres in the Largest fibre size cluster (*B* = 11 351, standardized *B* = 0.557, *P* < .001) were shown to be predictive of the overall increase in muscle fibre CSA over time. The overall model fit was *R* ^2^ = 0.684, *P* < .001. The increase in the percentage of muscle fibres in the Largest muscle fibre size cluster was the only significant predictor for the increase in myonuclear content in response to the exercise training programme (*B* = 5.166, standardized *B* = 0.616, overall model fit *R* ^2^ = 0.380, *P* < .001). In contrast, the decline in the percentage of muscle fibres in the Small fibre size cluster was the only significant predictor for the increase in myonuclear domain size in response to training (*B* = −1033, standardized *B* = −0.466, overall model fit *R* ^2^ = 0.217, *P* = .002).

## 3 DISCUSSION

The present study shows that prolonged resistance exercise training results in significant gains in lean tissue mass and strength, which is accompanied by muscle fibre hypertrophy and myonuclear accretion in healthy, older men. We observed that the small muscle fibres have a disproportionally small myonuclear domain size when compared with the larger muscle fibres. In addition, we show that exercise training‐induced muscle fibre hypertrophy is associated with a decline in the percentage of small and increase in percentage of the largest muscle fibres. This study is the first to show that the decline in the percentage of small muscle fibres following prolonged exercise training (with a disproportionally small myonuclear domain size) is best predicted by an increase in myonuclear domain size as opposed to an increase in myonuclear content in all muscle fibres. In contrast, the increase in the percentage of the largest muscle fibres in response to exercise training is best predicted by an increase in myonuclear content and not an increase in myonuclear domain size in these healthy, older men.

The myonuclear domain theory postulates that the ratio between myonuclear content and domain size is kept relatively constant under conditions of muscle fibre hypertrophy and atrophy.^6^ In accordance, we observed that muscle fibre hypertrophy was associated with myonuclear accretion and show a (strong) positive linear relationship between muscle fibre CSA and myonuclear content (Figure S1A). These observations are in line with other in vivo human studies performed at rest or in response to prolonged exercise training.^24^, ^26^, ^29^, ^30^, ^31^, ^32^ Furthermore, data from our laboratory suggest that *large* changes in domain size do not occur (or persist) under physiological conditions like muscle fibre hypertrophy during prolonged exercise training.^20^ However, this does not necessarily imply that myonuclear domain size is not flexible, especially since other studies have reported that exercise‐induced muscle fibre growth can occur without myonuclear accretion, but rather increasing myonuclear domain size in young and older individuals.^33^, ^34^, ^35^ These discrepancies may, in part, be explained by the way studies assess the changes in muscle fibre CSA, myonuclear content and domain size, as they typically report mean changes in the entire muscle biopsy cross‐section analysed.^8^, ^23^ Importantly, recent studies have shown that myonuclear domain sizes differ extensively between clusters of different muscle fibre sizes.^25^, ^26^, ^27^ In line with previous publications,^26^, ^27^ we show that myonuclear domain size is disproportionally small, in small (2000‐3999 µm^2^) compared with larger (4000‐9999 µm^2^) muscle fibres within the same muscle biopsy sample. Although the origin of the relatively large percentage (23 ± 17%) of small muscle fibres observed in the muscle biopsy samples at baseline cannot be verified within the current study design, based on previous studies ^24^, ^36^, ^37^, ^38^, ^39^, ^40^, ^41^ as well as the age of the included participants (70 ± 3 years), it is likely that many fibres have atrophied prior to our subjects reaching their present age. This seems to be supported by the significant (weak‐to‐moderate) negative correlation between the percentage of small muscle fibres and Quadriceps muscle CSA as well as 1RM muscle strength. Although cross‐sectional studies suggest that the age‐related decrease in mean muscle fibre CSA is accompanied by a mean reduction in myonuclear content,^24^, ^39^, ^40^, ^41^ the relatively small myonuclear domain size of the smaller fibres may suggest that the decline in muscle fibre size proceeds at a faster rate than the concomitant decline in myonuclear content.^24^ It has been postulated that having a relatively high number of myonuclei for its fibre size may also be beneficial, as regrowth of these muscle fibres may not require the generation of de novo myonuclei from the satellite cell pool.^14^, ^15^ Yet, on the other hand, ageing may also be accompanied by a reduced myonuclear efficiency equally reflected by a lower myonuclear domain size in the smallest (atrophied) muscle fibres.^42^ If myonuclear efficiency is lost to such a degree that the muscle fibre atrophies, it may result in a greater need to incorporate additional myonuclei to facilitate regrowth of these muscle fibres in response to anabolic stimuli in older individuals. Therefore, investigating the changes in myonuclear domain size within different muscle fibre size clusters could provide more insight in the regulation of myonuclear content and the proposed necessity of myonuclear accretion during muscle fibre hypertrophy following prolonged exercise training.

The current study shows that a greater decrease in the percentage of the smallest fibres and a greater increase in the percentage of the largest muscle fibres are associated with greater increases in overall muscle fibre size. This suggests that even the smaller muscle fibres from these senescent muscle tissue samples are still able to increase in size in response to prolonged resistance exercise training. Interestingly, we show that the decline in the percentage of small muscle fibres (with a disproportionally small myonuclear domain size) was best predicted by an overall increase in myonuclear domain size and not by an increase in myonuclear content as assessed in the entire muscle biopsy sample following exercise training. This suggests that the existing myonuclei located in the small muscle fibres are able to support the exercise training‐induced muscle fibre hypertrophy without the need to generate additional myonuclei. In other words, the smaller muscle fibres in aged muscle tissue contain ample functional myonuclei which are able to support muscle fibre growth in response to prolonged resistance exercise training. In contrast, the exercise‐induced increase in the percentage of the largest muscle fibres (with a myonuclear domain size around 2300 µm^2^) is best predicted by an overall increase in myonuclear content as opposed to an increase in domain size. This appears to be in line with the myonuclear domain ceiling theory, which suggests that myonuclear addition is prerequisite beyond a certain myonuclear domain size threshold.^12^, ^13^ Altogether, these data indicate that the myonuclear domain size is flexible, varies between muscle fibre size clusters, and that the size of the muscle fibre and in association the size of its myonuclear domains likely dictates the necessity for myonuclear addition to allow further fibre growth following prolonged exercise training in older individuals.

Skeletal muscle satellite cells are the only known source to provide additional myonuclei to support muscle fibre hypertrophy. A number of studies have reported an increase in resting muscle satellite cell content in response to prolonged resistance exercise training.^12^, ^13^, ^20^, ^21^, ^23^, ^35^, ^43^ In addition, a positive relationship has been shown between the increase in resting muscle satellite cell content and the extent of muscle fibre hypertrophy during exercise training.^13^, ^32^, ^41^ In the present study, resting muscle satellite cell content remained unchanged over time, also when expressed for type I and type II muscle fibres separately (data not shown). Muscle satellite cell content is reduced in older adults,^41^ however, this has previously not limited our,^8^, ^21^, ^41^ as well as other^12^, ^23^ laboratories to show a systemic increase in satellite cell content in response to exercise training in older adults.^12^, ^13^, ^35^ Despite the fact that we did not detect a significant increase in satellite cell content in response to exercise training, we did observe a significant (but weak) positive correlation between the increase in satellite cell and myonuclear content. In contrast, no significant correlations were observed between the change in satellite cell content following exercise training and the changes in the percentage of muscle fibres in any of the muscle fibre size clusters. It could be speculated that the lack of change in muscle satellite cell content following exercise training signifies an impaired muscle reconditioning response with age. Alternatively, as overall myonuclear content had increased following the training period, satellite cell content may have already returned to baseline levels, after an initial increase earlier on in the training, as a result of the subsequent proliferation, differentiation and fusion of satellite cell progeny with the existing muscle fibres. In any case, the exact role of (impaired) satellite cell function in determining the hypertrophic response to exercise training with ageing, including the potentially differential role in fibres of different size, remains to be further determined.

Since we included healthy older adults only, it remains to be established whether similar results would be obtained in young adults in response to prolonged exercise training. Previously, Karlsen et al^26^ have shown that small muscle fibres (1000‐3000 µm^2^) are characterized by a similar disproportionally small myonuclear domain size in both young and older subjects. It has been speculated that these smaller muscle fibres hold a greater growth potential when compared with larger muscle fibres.^27^ However, the proportion of small muscle fibres at baseline is far greater in senescent compared with younger muscle tissue,^24^, ^26^ which may limit verification of the current study results in younger participants. Furthermore, it is important to note that in the current study, older adults were included who were generally in good health and were still living independently. Recently, Karlsen et al^27^ have shown that the muscle fibre hypertrophy response may be substantially reduced in more frail, older adults (83‐94 y). A reduction in myonuclear and/or satellite cell function at these later stages of life will likely change the dynamics between myonuclear domain size and the potential need for myonuclear accretion. The original intention of the present study was to evaluate all muscle biopsy outcome parameters in a fibre type‐specific manner, as evidenced by the fibre type‐specific stain that was performed. Although the cluster analysis may provide a more sensitive approach to establish differential responses between muscle fibres of different sizes, it also requires quite a large muscle biopsy sample to include sufficient muscle fibres per cluster to make a reliable estimation of myonuclear content and domain size per cluster. The number of muscle fibres per biopsy sample turned out to be too small to assess the changes over time within each muscle fibre cluster in type I and type II muscle fibres separately and, as such, all muscle biopsy data are presented for mixed muscle fibre type. The lack of fibre type‐specific data is clearly a limitation of the present study, especially when considering that type II muscle fibres are specifically affected by ageing and resistance exercise training.^41^ Presenting the data mixed for muscle fibre type did allow us to include a fourth cluster, in this case the largest muscle fibre size cluster (8000‐9999 µm^2^), which has been key to provide evidence for the suggestion of the observed differential myonuclear accretion responses between the different muscle fibre size clusters. Nevertheless, it would be of interest to observe whether the observed changes and associations are driven by a specific muscle fibre type. Although the muscle fibre cluster approach does provide additional insight, it also holds limitations. The lack of change in fibre percentage in the Moderate and Large muscle fibre size clusters in response to exercise training does not mean that these muscle fibres are not growing. The growth of a particular muscle fibre can surpass the upper limit of its fibre size cluster, thereby “transferring” it into a larger muscle fibre size cluster. It could very well be that in the Moderate and Large muscle fibre clusters, just as many fibres are moving in from a smaller cluster and moving out towards a larger cluster resulting in no change in the percentage of the fibres in that particular cluster, and as such, no association with the overall increase in muscle fibre size of all muscle fibres. Finally, the present approach assumes that myonuclei are also homogeneously positioned throughout the muscle fibres. However, Cristea et al^36^ elegantly showed that the spatial orientation of myonuclei also changes with increasing age, potentially reflecting an age‐related loss in myonuclear efficiency, and this is not accounted for in this study. In future studies it would of interest to assess whether the spatial orientation of myonuclei is more distorted in the relative small compared with the larger muscle fibres and how this related to the muscle fibre growth response within older adults.

In conclusion, we provide evidence that both small as well as large muscle fibres contribute to overall muscle hypertrophy that is observed in response to prolonged resistance exercise training in healthy, older adults. This study shows that the increase in the percentage of small muscle fibres is best predicted by an increase in overall myonuclear domain size and not myonuclear number, whereas increase in the percentage of the largest muscle fibres is accompanied by an increase in overall myonuclear content and not domain size. These findings increase our insight in the dynamics between myonuclear domain size and myonuclear accretion following exercise training‐induced muscle fibre hypertrophy.

## 4 MATERIEL AND METHODS

### 4.1 Participants

Forty, healthy older men (70 ± 3 years; 78 ± 8 kg; 25 ± 2 kg/m^2^) participated in a 12‐week resistance exercise training programme. Participants were screened for medical issues, and excluded if any gastrointestinal, cardiovascular, neurological or renal diseases were present. Fasting blood glucose concentrations and glycated haemoglobin contents were assessed to screen for the presence of type 2 diabetes. All participants lived independently and had not been participating in any structured, progressive resistance exercise training programme within the past 3 years. All of the subjects were informed of the nature and possible risks of the experimental procedures before their written informed consent was obtained. The study was approved by the Medical Ethical Committee of the Maastricht University Medical Centre+ (METC 15‐3‐003) and conformed to the standards for the use of human subjects in research as outlined in the most recent version of the Helsinki Declaration. This study was part of a larger research project investigating the impact of protein supplementation on the gains in muscle mass and strength during resistance exercise training in healthy, older men, for which the main study outcomes have been published previously.^28^ As no differences in the gain in muscle mass and strength were observed with or without protein supplementation during the exercise training programme,^28^ all participants were considered as one group within this study.

### 4.2 Exercise training programme

The supervised resistance exercise training programme was performed three times per week for 12 weeks, as described previously.^28^ In short, following a 5 minutes warm‐up on a cycle ergometer, four sets were performed on both the leg press and knee extension machines (Technogym, Rotterdam, the Netherlands). Upper body exercises were paired (chest press with lateral pulldown and shoulder press with horizontal row) and were performed in an alternating manner between training sessions, with 2 sets of each exercise performed. During the first 4 weeks of training, the workload was increased from 70% 1RM (8 repetitions in each set) to 80% 1RM (10 repetitions). In addition, the workload was increased when >10 repetitions could be performed. Resting periods of 1.5 and 3 minutes were allowed between sets and exercises respectively. Participants were personally supervised throughout the exercise training programme. Each exercise session ended with a 5 minutes cool‐down on a cycle ergometer.

### 4.3 Body composition

Body composition was assessed at the whole‐body and regional level using Dual‐Energy X‐ray Absorptiometry (DXA; Discovery A, QDR Series; Hologic, Bradford, MA). Anthropometric measurements were assessed using standardized procedures, body mass by a digital scale to within 100 g and height by a stadiometer to within 0.5 cm. Anatomic cross‐sectional area (CSA) of the quadriceps muscle was assessed by computed tomography scanning (Philips Brilliance 64; Philips Medical Systems) before and after the 12‐week exercise training intervention, as described previously.^28^

### 4.4 Muscle strength assessment

Maximum strength was assessed by 1‐RM strength tests on leg press and knee extension machines (Technogym). During a familiarization trial, proper lifting technique was demonstrated and practiced and maximum strength was estimated. In an additional session, at least 1 week before muscle biopsy collection, each subject's 1 RM strength was determined as described previously.^44^

### 4.5 Muscle biopsy sampling

Muscle biopsies were taken from the right leg of each subject in the morning after an overnight fast. The post‐training muscle biopsy was taken at least 4 days after the final exercise session. The incision of the post‐training muscle biopsy was at least 3 cm proximal from the pre‐training biopsy sampling. After local anaesthesia was induced, percutaneous needle biopsy samples (50‐80 mg) were collected from the *vastus lateralis* muscle, approximately 15 cm above the patella.^45^ Any visible non‐muscle tissue was removed immediately, and biopsy samples were embedded in Tissue‐Tek (Sakura Finetek, Zoeterwoude, the Netherlands), frozen in liquid nitrogen‐cooled isopentane and stored at −80°C until further analyses.

### 4.6 Immunohistochemistry

**FIGURE 4:**
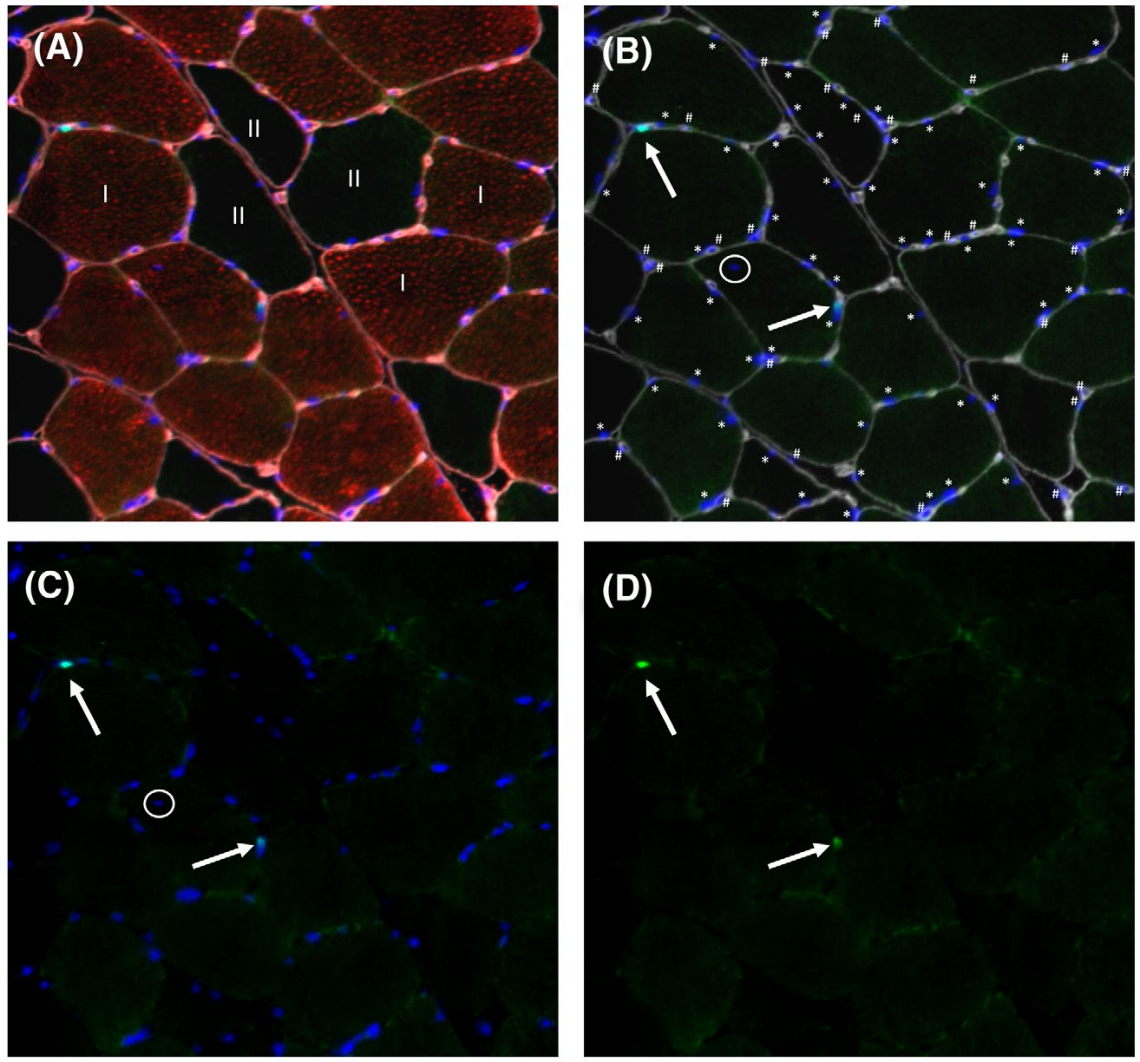
Representative image of the immunohistological staining of muscle fiber size, myonuclear/satelite cell content and myonuclear domain size. A, Laminin in grey, Pax7 in green, Myosin Heavy Chain (MHC) I in red and DAPI in Blue. B, Pax7 in green, Myosin Heavy‐Chain (MHC) I in red, DAPI in Blue. C, Pax7 in green, DAPI in Blue. D, Pax7 in green only. I: type I muscle fiber. II: type II muscle fiber. *Indicate DAPI+ cell within laminin boundaries; a myonucleus. ^#^Indicates DAPI+ cell outside the laminin boundary. Arrows point at DAPI+ Pax7+ (satellite) cell. Encircled DAPI+ cell: central myonucleus

From all biopsies, 5‐μm‐thick cryosections were cut at −20°C. Care was taken to properly align the samples for the cross‐sectional muscle fibre analyses. Muscle biopsies were stained to determine muscle fibre size, myonuclear and satellite cell content. In short, samples were air dried for 30 minutes after taking them out of the freezer. Samples were first fixated by a 5 minutes acetone incubation (VWR Chemicals, Vienna, Austria), followed by a 30 minutes blocking step in 3% BSA in 0.1% Tween/phosphate‐buffered saline (PBS). Between incubations, slides were rinsed 1 time with 0.1% Tween/PBS and 2 times with PBS for 5 minutes. The following antibodies were dissolved in a 0.1% BSA/0.1% Tween/PBS staining solution. Next, slides were first incubated overnight at 4°C with Pax7 (neat; cell supernatant from cells obtained from the DSHB, Iowa City, IA; kindly provided by G. Parise). On the second day, slides were incubated for 30 minutes with HAM Biotin (BA‐2000, Vector Laboratories, Burlingame, CA, 1:200). Next, samples were incubated for 30 minutes with Streptavidin Alexa 488 (S32354, Invitrogen, Grand Island, NY, 1:200), anti‐myosin heavy‐chain type 1 (A4.840, DSHB, 1:25) and anti‐Laminin (L9393, Sigma‐Aldrich, Darmstadt, Germany, 1:50). Appropriate secondary antibodies were applied, GAMIgM Alexa 555 (A21426, Invitrogen, 1:500) and GARIgG Alexa 647 (A21238, Invitrogen, 1:400) in combination with DAPI (0.238 μmol/L; Life Technologies) for 30 minutes. Finally, slides were mounted with Mowiol (Calbiochem, Amsterdam, The Netherlands) afterwards and covered by a glass coverslip. The staining procedure resulted in laminin staining grey, MHCI red, Pax7 green and DAPI blue (Figure 4). Images were visualized and automatically captured with a 10× objective (110x magnification) with a fluorescent microscope equipped with an automatic stage (IX81 motorized inverted microscope; Olympus, Hamburg, Germany). Quantitative analyses were performed using ImageJ software package (version 1.46d, National Institute of Health, MD). For muscle fibre CSA, laminin was used to automatically detect the outline of the individual muscle fibre; corrections were made by hand where necessary. Fibber CSA was automatically determined for each muscle fibre. Myonuclei enumerations were performed semi‐automatically, corrections were made by hand where necessary. DAPI+ cells were automatically detected by the ImageJ macro and were only counted when at least 50% at the DAPI+ cell was located within the laminin outline to determine myonuclear content per individual muscle fibre, which was corrected for the number of satellite cells (ie only nuclei within cell border and not expressing Pax7 were counted as myonuclei). Centrally located nuclei (DAPI+/Pax7‐ cell located within the boundaries but detached from the laminin) were excluded from the myonuclear counts. Satellite cell enumerations were performed manually. A muscle satellite cell was identified as a DAPI+/Pax7+ cell located within the muscle fibre (ie laminin staining). All image recordings and analyses were performed by an investigator blinded to participant coding. An average number of 412 ± 251 and 321 ± 171 muscle fibres were included per biopsy sample to establish mean muscle fibre CSA, myonuclear content, domain size and satellite cell content before and after 12 weeks of resistance exercise training respectively.

### 4.7 Muscle fibre size cluster analyses

To compare myonuclear content and domain size in muscle fibres of similar size before and after 12 weeks of resistance exercise training, all cross‐sectional muscle fibres were ordered by size and four muscle fibre size clusters were created: ‘Small’: 2000‐3999 µm^2^, ‘Moderate’: 4000‐5999 µm^2^, ‘Large’: 6000‐7999 µm^2^ and ‘Largest’: 8000‐9999 µm^2^. The size of each cluster was based on previous publications.^26^, ^27^ Owing to the small number of subjects with fibres smaller than 2000 µm^2^ and/or larger than 9999 µm^2^, these fibres were excluded from the fibre cluster analysis, resulting in an average of 91 ± 12% of all muscle fibres from the pre‐training biopsies, and 88 ± 11% of all muscle fibres from the post‐training biopsies that were included within the muscle fibre cluster analyses. The percentage of muscle fibres per fibre size cluster was calculated as total number of cross‐sectional fibres within a fibre size cluster divided by the total number of cross‐sectional fibres of the entire muscle cross‐section· (100%). In addition, the change in the percentage of fibres per fibre size cluster from pre‐ to post‐exercise training was calculated as the percentage of fibres per muscle fibre size cluster post training minus the percentage of fibres per muscle fibre size cluster pre‐training. For accurate assessment of myonuclear content and domain size pre‐ and post‐training, a minimum of 10 muscle fibres per fibre size cluster was required to include participant in the correlation analyses.^26^, ^27^ Myonuclear domain per fibre size cluster was calculated by dividing the average size of the fibres within one cluster by the average number of myonuclei per fibre within the cluster. See Table 2 for an overview of the total number of participants and average number of muscle fibres included for myonuclear content and domain size per fibre size cluster pre and post 12 weeks of resistance exercise training.

**TABLE 2:** Number of participants and fibres included in the cluster analysis for myonuclear content and domain size before (pre) and after (post) 12 wk of resistance exercise training in healthy, older men

	Pre		Post	
	Participants (n)	Number of fibres	Participants (n)	Number of fibres
Small cluster	39	107 ± 120	34	81 ± 107
Moderate cluster	40	133 ± 75	39	110 ± 72
Large cluster	38	89 ± 42	40	82 ± 41
Largest cluster	26	46 ± 25	37	50 ± 27

Note

Total number of participants with ≥10 fibres within the 2000 µm^2^ cluster and mean number of fibres (±SD) per participant within each cluster. Small: muscle fibres within size of 2000 and 3999 µm^2^. Moderate: muscle fibres within size of 4000 and 5999 µm^2^. Large: muscle fibres within size of 6000 and 7999 µm^2^. Largest: muscle fibres within size of 8000 and 9999 µm^2^.

### 4.8 Statistics

All data are expressed as mean ± standard deviation. Normal distribution of all parameters was verified by Kolmogorov‐Smirnov test. A paired samples t‐test was used to evaluate the effect of training on 1RM muscle strength, body composition and mean muscle fibre size (pre vs post). A one‐way ANOVA was used to evaluate differences in myonuclear content and domain size between the different muscle fibre size clusters pre‐training (Small vs Moderate vs Large vs Largest). For the muscle fibre size cluster data, resistance exercise training‐induced changes were analysed with a repeated measures ANOVA with time (pre vs post) and fibre size cluster (Small vs Moderate vs Large vs Largest) as within subject factors. In case of a significant main effect of time or *time* × *fibre size cluster* interaction, pairwise comparisons with Bonferroni correction were performed to locate the significant differences. Pearson correlation analysis (*r*) was applied to evaluate the linear relationship between the change (∆) in muscle fibre cross‐sectional, ∆ myonuclear content, ∆ myonuclear domain size, Quadriceps CSA, 1RM muscle strength and ∆ muscle fibre size cluster (Small vs Moderate vs Large vs Largest). A significant r‐value between 0 and 0.19 was regarded as ‘very weak’, between 0.2 and 0.39 as ‘weak’, between 0.40 and 0.59 as ‘moderate’, between 0.6 and 0.79 as ‘strong’ and between 0.8 and 1 as ‘very strong’ correlation. Backward stepwise linear regression modelling was used to identify which ∆ muscle fibre size cluster (Small, Moderate, Large and/or Largest) is predictive of the overall mean change in muscle fibre CSA, myonuclear content and myonuclear domain size in response to the exercise intervention programme. Significance was set at *P* < .05. All calculations were performed using SPSS version 22.0 (SPSS version 22.0, IBM Corp., USA).

## CONFLICT OF INTEREST

The authors declare that they have no conflict of interest.

## Supporting information

Fig S1

Fig S2

Table S1‐S5
