# Supplementary material for: Myonuclear content and domain size in small versus larger muscle fibres in response to 12 weeks of resistance exercise training in older adults
Source: Acta Physiol (Oxf). 2020 Dec 20;231(4):e13599. doi: 10.1111/apha.13599 (PMC8047909; doi:10.1111/apha.13599)
Supplement: Supplementary file 1 — Fig S1 [file APHA-231-e13599-s002.pdf]

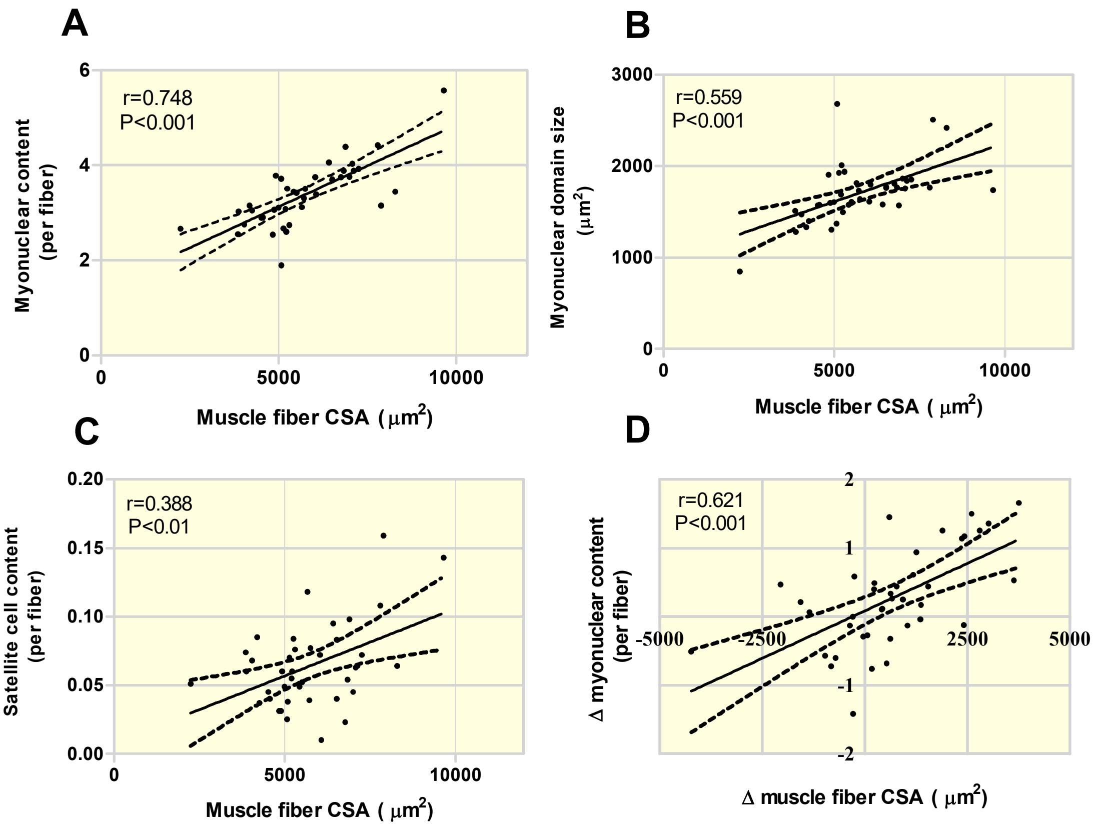

Supplemental figure 1: Scatter plots of the correlation analyses (Pearson's r, n=40) between baseline muscle fiber cross-sectional area (CSA) and myonuclear content (A), myonuclear domain size (B) and satellite cell content (C) in healthy, older men. Correlation between the changes in muscle fiber CSA and myonuclear content in response to 12 weeks of resistance exercise training (D). Solid line: regression line. Dotted line: 95% confidence interval.
