# Supplementary material for: Myonuclear content and domain size in small versus larger muscle fibres in response to 12 weeks of resistance exercise training in older adults
Source: Acta Physiol (Oxf). 2020 Dec 20;231(4):e13599. doi: 10.1111/apha.13599 (PMC8047909; doi:10.1111/apha.13599)
Supplement: Supplementary file 2 — Fig S2 [file APHA-231-e13599-s003.pdf]

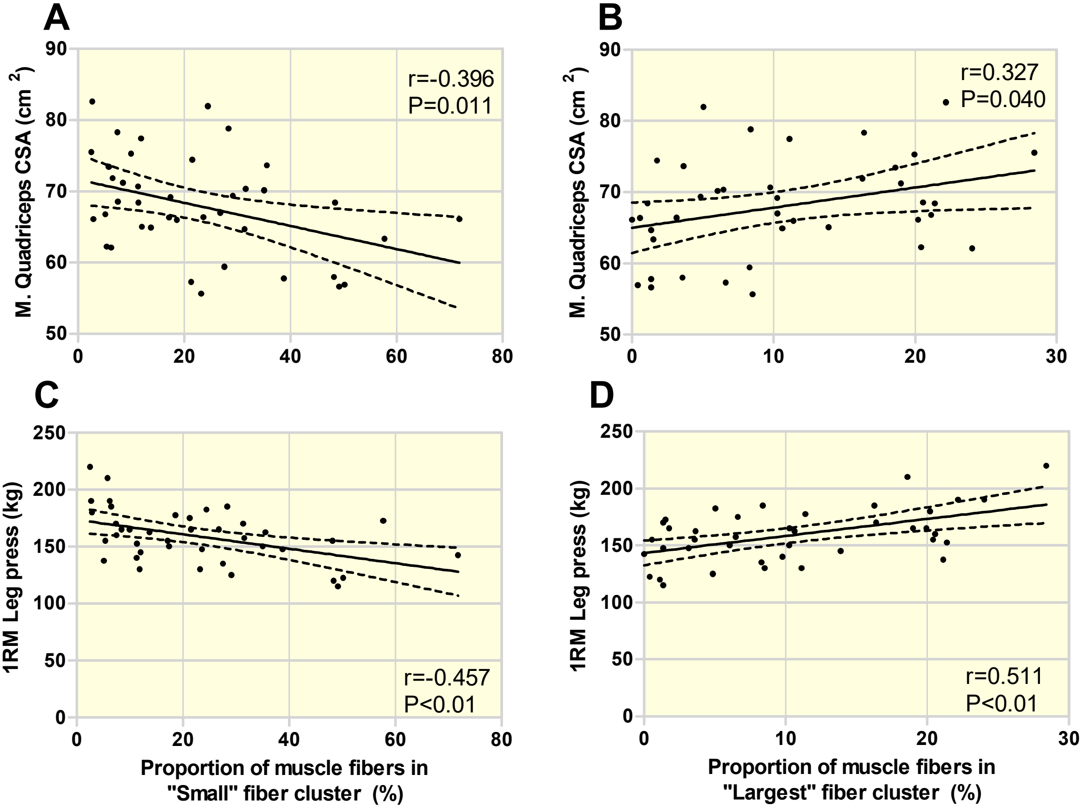

Supplemental figure 2: Scatter plots of the correlation analyses (Pearson's r, n=40) between the change in muscle Quadriceps cross-sectional area (CSA) (A-B) and 1RM leg press (C-D) and proportion (%) of muscle fibers within the "Small" (2000-3999 µm2) or "Largest" (8000-9999 µm2) muscle fiber size cluster in healthy older men. Solid line: regression line. Dotted line: ninety-five confidence interval.
