# Supplementary material for: Myonuclear content and domain size in small versus larger muscle fibres in response to 12 weeks of resistance exercise training in older adults
Source: Acta Physiol (Oxf). 2020 Dec 20;231(4):e13599. doi: 10.1111/apha.13599 (PMC8047909; doi:10.1111/apha.13599)
Supplement: Supplementary file 3 — Table S1‐S5 [file APHA-231-e13599-s001.docx]

**Supplemental tables**

| **Supplemental table 1. Muscle fiber characteristics in the placebo and protein supplemented group before (pre) and after (post) 12 weeks of resistance exercise training in healthy, older men.** | | | | |  |
| --- | --- | --- | --- | --- | --- |
|  | **Pre** | | **Post** | |  |
|  | **Placebo** | **Protein** | **Placebo** | **Protein** | |
| Muscle fiber CSA (µm^2^) | 5714±1139 | 5778±1648 | 6205±1468* | 6588±1597* | |
| Myonuclear content (per fiber) | 3.28±0.58 | 3.48±0.73 | 3.37±0.51* | 3.92±0.74* | |
| Myonuclear domain size (µm^2^) | 1764±340 | 1649±309 | 1846±369 | 1693±333 | |
| Satellite cell content (per fiber) | 0.055±0.021 | 0.065±0.021 | 0.074±0.037 | 0.074±0.035 | |
| Data represent mean ± SD. CSA: cross-sectional area. * significantly different compared with Pre, P<0.05. Participants were randomly divided to consume either 21 g leucine-enriched whey protein (3 g total leucine) (Protein) or an energy-matched placebo (Placebo) after exercise and each night before sleep, including rest days during the 12 weeks resistance exercise training program, see also Holwerda *et al.* ^28^ | | | | | |

| **Supplemental Table 2: the muscle fiber percentage, myonuclear content and domain size per muscle fiber size cluster before (pre) and after(post) 12 weeks of resistance exercise training in older adults.** | | | |
| --- | --- | --- | --- |
|  | **Fiber cluster** | **Pre** | **Post** |
| Fiber percentage (%) | Small | 23±17 | 17±14* |
|  | Moderate | 34±11 | 31±12 |
|  | Large | 24±11 | 26±9 |
|  | Largest | 11±8 | 15±10* |
| Muscle fiber size (µm^2^) | Small | 3040±355 | 3050±479 |
|  | Moderate | 4966±169 | 5030±159 |
|  | Large | 6573±991 | 6752±472 |
|  | Largest | 8452±750 | 8105±1567 |
| Myonuclear content (per fiber) | Small | 2.50±0.39 | 2.66±0.61 |
|  | Moderate | 3.23±0.40 | 3.30±0.56 |
|  | Large | 3.98±0.45 | 4.08±0.69 |
|  | Largest | 4.76±0.64 | 4.68±0.87 |
| Myonuclear domain (µm^2^) | Small | 1241±235 | 1257±285 |
|  | Moderate | 1561±200 | 1564±258 |
|  | Large | 1683±335 | 1698±291 |
|  | Largest | 1867±298 | 1833±473 |
| *Data are expressed Mean ± SD.* Small: muscle fibers within size of 2000 and 3999 µm^2^. Moderate: muscle fibers within size of 4000 and 5999 µm^2^. Large: muscle fibers within size of 6000 and 7999 µm^2^. Largest: muscle fibers within size of 8000 and 9999 µm^2^. *: significantly different compared with pre, P < 0.05. | | | |

| **Supplemental table 3**: Linear model of predictors of muscle fiber hypertrophy following 12 weeks of resistance exercise training, based upon backward linear regression analyses. | | | | | |
| --- | --- | --- | --- | --- | --- |
|  |  | b (95 % CI) | Coefficients SE | ß | p-value |
| *1* | *Constant* | -77 (-410 – 256) | 164 |  | 0.643 |
|  | Small cluster | -5327 ( -8562 – -2092) | 1594 | -0.570 | **0.002** |
|  | Moderate cluster | -612 ( -3896 – 2671) | 1617 | -0.054 | 0.707 |
|  | Large cluster | -3319 (-7658 – 1021) | 2137 | -0.233 | 0.130 |
|  | Largest cluster | 9641 (2991 – 16290) | 3276 | 0.490 | **0.006** |
| *2* | *Constant* | -82 ( -409 – 246) | 162 |  | 0.615 |
|  | Small cluster | -5152 (-8208 – -2096) | 1507 | -0.551 | **0.002** |
|  | Large cluster | -3645 ( -7564 – 274) | 1932 | -0.256 | 0.067 |
|  | Largest cluster | 10524 (5916 – 15133) | 2272 | 0.535 | **<0.001** |
| *3* | *Constant* | -60 (-397 – 278) | 167 |  | 0.722 |
|  | Small cluster | -3133 ( -5355 – -911) | 1097 | -0.335 | **0.007** |
|  | Largest cluster | 11350 (6680 – 16022) | 2305 | 0.577 | **<0.001** |
| Note: Model 1: R^2^ : 0.714. Model 2: R^2^ : 0.713. Model 3: R^2^ : 0.684. Small: muscle fibers within size of 2000 and 3999 µm^2^. Moderate: muscle fibers within size of 4000 and 5999 µm^2^. Large: muscle fibers within size of 6000 and 7999 µm^2^. Largest: muscle fibers within size of 8000 and 9999 µm^2^ | | | | | |

| **Supplemental table 4**: Linear model of predictors of myonuclear accretion following 12 weeks of resistance exercise training, based upon backward linear regression analyses. | | | | | |
| --- | --- | --- | --- | --- | --- |
|  |  | b (95 % CI) | Coefficients SE | ß | p-value |
| *1* | *Constant* | 0.037 (-0.177 – 0.250) | 0.105 |  | 0.730 |
|  | Small cluster | 0.164 (-1.910 – 2.238) | 1.022 | 0.039 | 0.873 |
|  | Moderate cluster | -1.419 (-3.523 – 0.686) | 1.037 | -0.278 | 0.180 |
|  | Large cluster | 1.913 (-0.868 – 4.695) | 1.370 | 0.297 | 0.171 |
|  | Largest cluster | 3.603 (-0.659 – 7.866) | 2.100 | 0.405 | 0.095 |
| *2* | *Constant* | 0.036 (-0.174 – 0.246) | 0.104 |  | 0.730 |
|  | Moderate cluster | -1.467 (-3.452 – 0.518) | 0.979 | -0.287 | 0.143 |
|  | Large cluster | 1.802 ( -0.564 – 0.518) | 1.167 | 0.280 | 0.131 |
|  | Largest cluster | 3.406 (-0.001 – 6.814) | 1.680 | 0.383 | 0.050 |
| *3* | *Constant* | 0.21 (-0.192 – 0.233) | 0.105 |  | 0.845 |
|  | Large cluster | 0.638 ( -1.155 – 2.431) | 0.885 | 0.099 | 0.476 |
|  | Largest cluster | 5.166 (2.689 – 7.642) | 1.222 | 0.581 | **<0.001** |
| *4* | *Constant* | 0.18 (-0.193 – 0.228) | 0.104 |  | 0.866 |
|  | Largest cluster | 5.166 (3.180 – 7.77) | 1.235 | 0.616 | **<0.001** |
| Note: Model 1: R^2^ : 0.425. Model 2: R^2^ : 0.424. Model 3: R^2^ : 0.389. Model 4: R^2^ : 0.380. Small: muscle fibers within size of 2000 and 3999 µm^2^. Moderate: muscle fibers within size of 4000 and 5999 µm^2^. Large: muscle fibers within size of 6000 and 7999 µm^2^. Largest: muscle fibers within size of 8000 and 9999 µm^2^ | | | | | |

| **Supplemental table 5**: Linear model of predictors of change in myonuclear domain size following 12 weeks of resistance exercise training, based upon backward linear regression analyses. | | | | | |
| --- | --- | --- | --- | --- | --- |
|  |  | b (95 % CI) | Coefficients SE | ß | p-value |
| *1* | *Constant* | -11 (-136 – 114) | 62 |  | 0.863 |
|  | Small cluster | -1343 (-2257 – -129) | 598 | -0.606 | **0.031** |
|  | Moderate cluster | 782 (-450 – 2014) | 607 | 0.292 | 0.206 |
|  | Large cluster | -1333 (-2962 – 296) | 802 | -0.395 | 0.106 |
|  | Largest cluster | 830 (-1667 – 3326) | 1230 | 0.178 | 0.504 |
| *2* | *Constant* | 2 (-116 – 120) | 58 |  | 0.970 |
|  | Small cluster | -1579 (-2556 – -602) | 482 | -0.712 | **0.002** |
|  | Moderate cluster | 491 (-367 – 1348) | 423 | 0.183 | 0.254 |
|  | Large cluster | -1245 ( -2837 – 348) | 786 | -0.369 | 0.122 |
| *3* | *Constant* | -12 (-127 – 104) | 57 |  | 0.840 |
|  | Small cluster | -1456 (-2412 – -500) | 472 | -0.656 | **0.004** |
|  | Large cluster | -867 (-2323 – 588) | 718 | -0.257 | 0.235 |
| *4* | *Constant* | -1 (-116 – 114) | 57 |  | 0.986 |
|  | Small cluster | -1033 (-1678 – -389) | 318 | -0.466 | **0.002** |
| Note: Model 1: R^2^:0.283. Model 2: R^2^:0.274. Model 3: R^2^:0.247. Model 4: R^2^:0.217 Small: muscle fibers within size of 2000 and 3999 µm^2^. Moderate: muscle fibers within size of 4000 and 5999 µm^2^. Large: muscle fibers within size of 6000 and 7999 µm^2^. Largest: muscle fibers within size of 8000 and 9999 µm^2^ | | | | | |
